# Supplementary material for: Non-Invasive Myocardial Work in Patients with Severe Aortic Stenosis
Source: J Clin Med. 2022 Jan 29;11(3):747. doi: 10.3390/jcm11030747 (PMC8836756; doi:10.3390/jcm11030747)

Supplementary Material

Supplementary Table S1. Echocardiographic data

	Pre-TAVI	Post-TAVI	P value
**Systolic BP (mmHg)**	135.4 ± 20.9	124.5 ± 14.5	0.002
**Diascolic BP (mmHg)**	74.7 ± 10.1	66.6 ± 8.7	<0.001
**TAPSE (mm)**	21.5 ± 3.1	23.7 ± 4	0.064
**PASP (mmHg)**	41.6 ± 10.9	39.7 ± 10	0.292
**LAVi (ml/m^3)**	43.8 ± 8.4	44.1 ± 8.8	0.341
**E/e‘**	13.2 ± 3	12.7 ± 2.1	0.334
**LVEDD (mm)**	50.6 ± 7.1	48.4 ± 6	0.869
**IVSd (mm)**	14.1 ± 3	13.9 ± 3.1	0.217
**LVEF (%)**	52.8 ± 9.3	52.1 ± 9.3	0.063
**GLS (%)**	-16.8 ± 5	-18.8 ± 4	0.060
**AV mean gradient (mmHg)**	46.7 ± 15.8	10.5 ± 5.9	<0.001

BP, Blood Pressure; TAPSE, Tricuspid Annular Plane Excursion; PASP, Pulmonary Artery Systolic Pressure; LAVi, Left Atrial Volume index; LVEDD, Left Ventricular End-Diastolic Diameter; IVSd, Interventricular Septal Diameter diastolic; LVEF, Left Ventricular Ejection Fraction; GLS, Global Longitudinal Strain; AV, Aortic Valve.

Legend to Supplementary Figures

Supplementary Figure S1 (SF1). Bland-Altman plot showing good correspondence between estimated and invasively measured left ventricular (LV) pressure. SD=standard deviation.

Supplementary Figure S2 (SF2). Comparison of mean baseline LV Ejection Fraction (upper panel) and Global Longitudinal Strain (lower panel) between men and women.

Supplementary Figure S3 (SF3). Comparison of mean baseline LV Ejection Fraction (upper panel) and Global Longitudinal Strain (lower panel) between patients with normal high gradient severe aortic stenosis (HG) and low flow/low gradient severe aortic stenosis (LF-LG).

Supplementary Figure S1 (SF1).

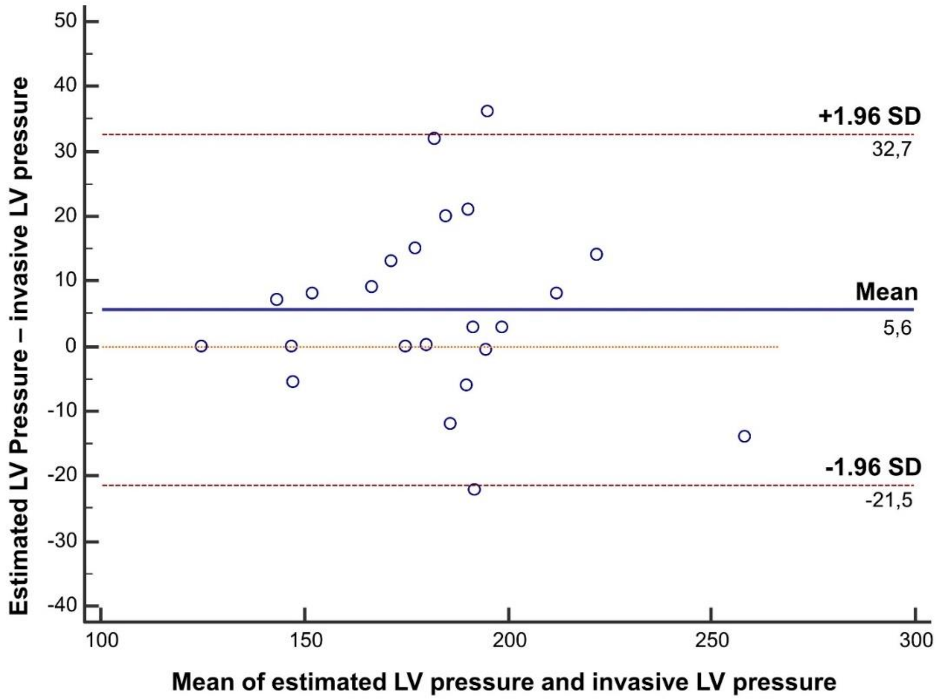

Supplementary Figure S2 (SF2).

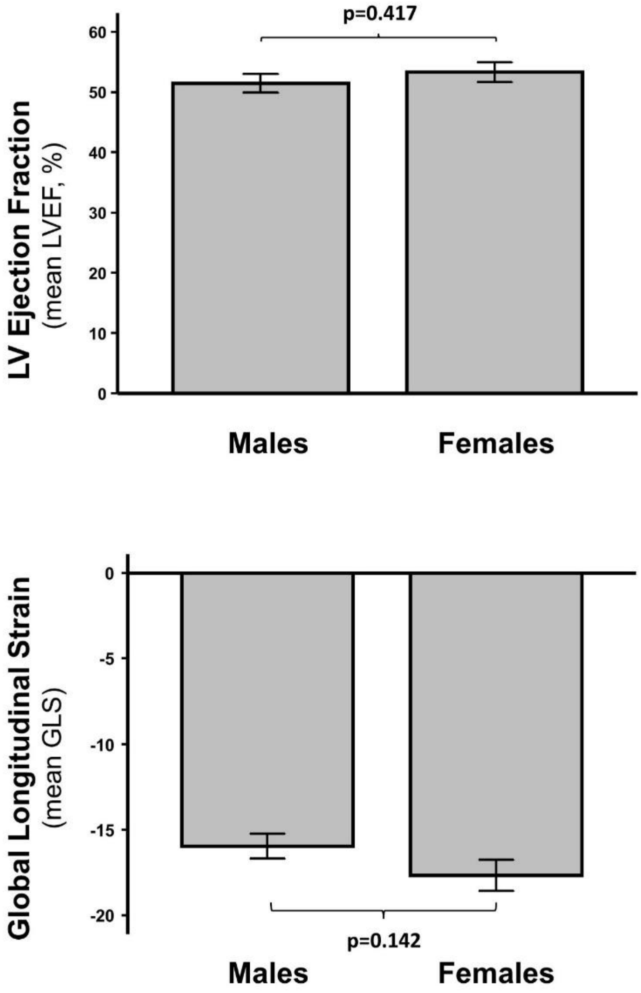

Supplementary Figure S3 (SF3).

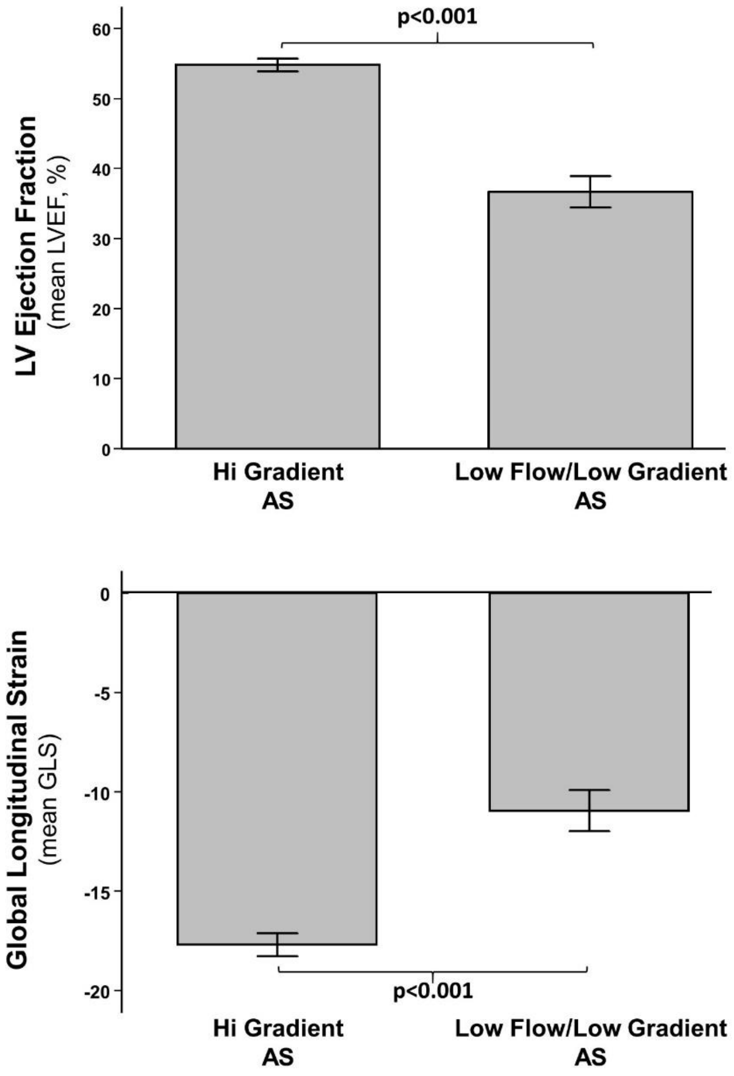

Supplement: Supplementary file 1 [file jcm-11-00747-s001.zip › jcm-1572942-supplementary.pdf]
